# Qualitative Risk Analysis for Contents of Dry Toilets Used to Produce Novel Recycling Fertilizers

**DOI:** 10.1007/s43615-021-00068-3

**Published:** 2021-07-15

**Authors:** Ariane Krause, Franziska Häfner, Florian Augustin, Kai M. Udert

**Affiliations:** 1 Leibniz Institute of Vegetable and Ornamental Crops (IGZ) e.V., program area ‘Next-Generation Horticultural Systems’ (HORTSYS), Großbeeren, Germany; 2 Finizio – Future Sanitation, Eberswalde, Germany; 3 ETH Zürich, Institute of Environmental Engineering, Zürich, Switzerland; 4 Eawag, Swiss Federal Institute of Aquatic Science and Technology, Dübendorf, Switzerland

**Keywords:** Human excreta, Compost, Urine, Sanitation, Pollutants, Pharmaceuticals, Nutrient cycling

## Abstract

Human excreta are a sustainable, economical source of nutrients, and can be used to produce recycling fertilizer for horticulture by collecting and processing the contents of dry toilets. Herein, we discuss the key categories of risk associated with the main groups of materials commonly found in dry toilets. The study was part of the development of a German product standard for marketable and quality-assured recycling fertilizers from human excreta for use in horticulture. Particular attention is paid to ensuring that the fertilizer is epidemiologically and environmentally harmless and that the quality of the recycling fertilizer is adequate in terms of low pollution and nutrient availability. In sum, the risk of transmissible human pathogens lies within the human excreta, particularly feces; plant materials added during composting are of particular phytosanitary relevance; pharmaceutical residues in excrements and chemical additives are potential sources of pollutants; non-biodegradable contaminants can cause pollution and injury; and the horticultural risks involve mainly the ammonia emission potential and in some cases the salinity effects of urine. These risks can be reduced significantly (i) with education of users around proper operation of dry toilets and the consequences of adding inappropriate waste, (ii) with facilitation of proper use with general waste bins and clear instructions, and importantly (iii) by using modern sanitization and cleaning processes and testing for harmful substances under the guidance of local laws and regulations, ensuring safe and high-quality fertilizers. In conclusion, the benefits of using dry toilet contents to produce fertilizers for use in horticulture are unquestionable. Our analysis highlights the need to support recycling optimization and awareness for the purpose of a sustainable circular economy and to minimize the risk of harm to humans and the environment overall.

## Introduction

**Fig. 1:**
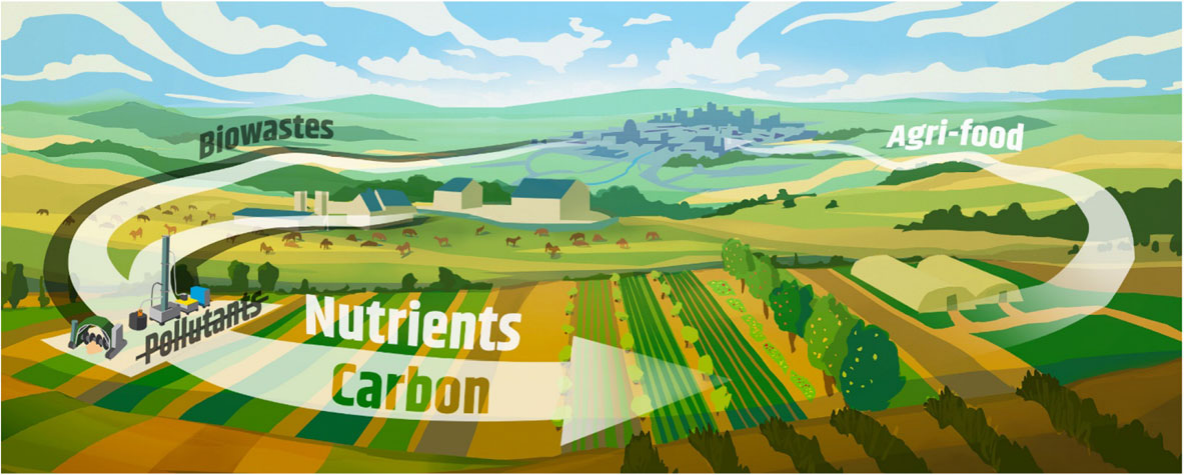
Vision of a regional CE based on an appropriate treatment of undiluted biowastes to integrate the removal of pollutants and pathogens with the recycling of nutrients and carbon for the production of safe and healthy agri-food (painted by Aaron Joao Markos, and licensed under CC BY-SA 4.0, available at www.naehrstoffwende.org)

In Europe, human excreta are almost exclusively discharged into wastewater, and nutrient recycling from human excreta is not yet part of a circular economy (CE) [1–3]. While animal excreta are an integral part of fertilization, substances of human origin are not recycled, although there is a clear fertilization potential because human excreta contain essential plant nutrients, such as phosphorus (P), nitrogen (N), or potassium (K) [1, 4–6]. Human urine and feces contribute 70–80% of N and up to 60% of P in urban municipal wastewater [7, 8]. In the sewage system, these nutrients are often contaminated with heavy metals and micro-plastic from sources other than toilets. Due to this contamination, field fertilization with sewage sludge has been restricted or banned by many national governments, including Germany [9]. Currently studied and applied processes for the recovery of nutrients from wastewater mainly focus on individual elements, e.g., P recovery by struvite precipitation from wastewater or P extraction from sewage sludge ash [10]. N is extracted from wastewater via nitrification and denitrification, which further leads to significant gaseous N losses due to nitrous oxide emissions from activated sludge processes [29]. So N is rather removed and not recycled. By means of decentralized collection, treatment and use of human urine and feces material flows [11, 12], regional, CE-orientated nutrient recycling can be achieved (Fig. 1).

The transformation of the sanitation sector from *disposal*- to *recycling*-centered is based on “renewable” technologies that (i) avoid the use of fresh water as flush water as far as possible, (ii) capture material flows separately where possible, and (iii) enable the recovery of nutrients [11, 13, 14]. The “material flow separation” specifically allows for the precise analysis of nutrients, pathogens, or pollutants (e.g., heavy metals or pharmaceutical/drug residues) and for the specific treatment of the undiluted materials [15, 16]. The aim of the treatment is to inactivate pathogens (“hygienization”) and eliminate pollutants in order to ultimately recycle the remaining nutrients safely [ibid.]. One common method of material separation “at the source” is the use of dry toilets (DTs), in which human excreta are collected without or very limited flushing [14, 17]. DTs are decentralized, modular, and flexible in use, and thus a core element of a sustainable, globally available sanitary technology [11]. Following the separation and collection of excreta from DTs, the treatment and recycling process begins [15, 16, 18]. Fertilizer products that can be produced from human excreta include (i) treated urine (e.g., stored [19, 20] or nitrified urine [21, 22], struvite [23–25]), (ii) fecal compost [26–28] or fermentation products [29, 30], or (iii) carbonized [31, 32] and/or pelletized [33] feces.

Over the last 10 years, countries such as Germany, Austria, and Switzerland have observed the development of a new market for mobile DTs for events, as well as stationary/permanently installed DTs for allotment gardens or mobile homes. The sustainability model of the involved start-ups and small- and medium-sized enterprises is characterized by the intention to reduce flush water use, to promote nutrient cycling, and to reject environmentally harmful chemicals that are often used in non-sewerage-bound sanitation. However, the development of the recent DT industry is currently being hampered by a number of administrative, legal, and economic uncertainties regarding recovery-oriented, soil-related treatment and the sales and use opportunities for the produced recycling fertilizers. In particular, the legal framework governing the marketing of fertilizers is currently an obstacle to making raw materials of anthropogenic origin usable as fertilizers [11, 34]. Existing laws, standards, and specifications only apply to recycling fertilizers that are produced from parent substrates of plant or animal origin, but not from anthropogenic parent substrates [35, 36]. Also at the European level, there is an absence of normative or legal regulations for recycling fertilizers from parent substrates of human origin [35, 37]. As a consequence, there is, to the best of our knowledge, currently no such recycling fertilizer on the German or Austrian market. One exception is a fertilizer made from human urine, which was approved in Switzerland in 2018 [38].

To sum up, in order to realize progressive regional CEs, there is a need to adapt and innovate existing fertilizer laws and regulations on a national and on a European level in order to integrate human excreta and/or content from DTs as potential parent substrates for the production of recycling fertilizers. Central to the debate on the potential horticultural application of such novel recycling fertilizers are the risks associated with DT contents. Hence, quality assurance plays a crucial role when setting the legal framework for the production and use of novel recycling fertilizers from DT contents. In this context, standardization can facilitate market deregulation by providing standardized procedures for such quality assurance [39]. In Germany, for example, the public sector sets the legal framework and lays down certain protection targets, e.g., for product, work, and environmental safety, when developing or adapting laws and regulations. Furthermore, these laws and regulations often refer to “generally accepted rules of technology” for reaching the protection targets. Hence, norms, standards, and technical rules, which give further details to legal obligations for fulfilling the protection targets, are supportive to the legislation process and, thus, to the public sector. Norms and standards can further serve as a benchmark to control and to compare the quality of products including of different recycling fertilizers. However, there is, to the best of our knowledge, no standard or product specification available that focuses on quality assurance for recycling fertilizer produced from human excreta or DT contents. Nevertheless, such document could help to (i) reduce existing uncertainties in the marketing of recycling fertilizers from human excreta, (ii) promote dialogue with policy makers, and (iii) establish sustainable, cycle-oriented sanitation systems and fertilizer alternatives.

To conclude, it was the objective of the present study to analyze the risks associated with the agricultural or horticultural use of recycling products from DT contents. This risk analysis can then serve to identify and describe specific requirements to characterize, assess, and assure the quality of recycled products as fertilizers.

## Methodology

This study is a qualitative risk analysis and is part of the development of the new standard for “quality assurance of recycling products from dry toilets for use in horticulture” (DIN SPEC 91421:2020) [40]. This product specification (DIN SPEC) was developed by a consortium of 18 experts in resource-based sanitation for the German Institute for Standardization (*Deutsches Institut für Normung*, DIN), and is intended to contribute to quality assurance in the establishment of regional CEs that involve human excreta in nutrient cycling practices. The product specification defines quality criteria and requirements for marketable and quality-assured recycling products from parent substrates of human origin for use as fertilizer in horticulture and is based on German and European legislation on fertilizers and recycling fertilizers. Concrete requirements for characteristics and properties of the recycling fertilizers were derived from the German fertilizer legislation (cf. “Definition of Quality Requirements and Associated Risk Categories Under Consideration” section).

The risk analysis is based on an extensive literature review. Moreover, risks are systematically considered for relevant material input flows associated with DT contents (cf. “Definition of the Scope of Material Flows Under Consideration” section) and discussed against certain risk categories defined as most relevant for marketable recycling fertilizers according to the German fertilization law (cf. “Definition of Quality Requirements and Associated Risk Categories Under Consideration”section). The risk analysis has a qualitative character, meaning that in the assessment, probability and consequence are not numerically estimated, but are evaluated verbally using qualifiers like high likelihood and low likelihood.

### Definition of the Scope of Material Flows Under Consideration

Since most important risks of recycling fertilizers are determined primarily by the quality and properties of the contents collected in DTs and any processing-related additive, the focus of this risk analysis is on risks due the quality of the input materials to a DT and how this can in turn influence the quality of the fertilizer products. For the purpose of this risk analysis, the contents of DTs, as potential source materials for fertilizer production, were categorized into three main groups of substances: (i) human excreta, composed of feces, urine, blood, and vomit; (ii) operational additives, including toilet-related additives (such as toilet paper, bedding material, or detergents) and recovery additives (e.g., for composting or urine treatment); and (iii) contaminants, including hygiene products, packaging waste, glass, and contents from chemical toilets.

It should be noted that these classes of materials are not always collected separately, but often occur in mixed forms (e.g., feces with toilet paper, urine with blood, or feces with urine and toilet paper). Even though separate collection can minimize the potential risks from the individual material flows and more efficiently provide added value, a complete separation of the substance groups is difficult to implement.

### Definition of Quality Requirements and Associated Risk Categories Under Consideration

According to the German Fertilizer Ordinance (*Düngemittelverordnung*, DüMV), quality assurance for recycled products must ensure their proper use [41]. Only fertilizers that “do not damage the fertility of the soil, the health of humans, animals and crops and do not endanger the balance of nature” (§ 3 I 1 DüMV) may be approved and placed on the market [ibid.]. Parent substrates (“*Ausgangsstoffe*”) used for fertilizer production must also comply with this specification and must have “a plant-growing, production or application-related benefit or serve to protect the soil and to maintain and promote soil fertility” (§ 3 I 2 DüMV) [ibid.]. Furthermore, only fertilizers that “do not contain pathogens, toxins or pests” may be approved and placed on the market (§ 5 I DüMV) [ibid.].

Therefore, the following four risk categories are considered most relevant for the horticultural applications of recycled products from DTs and are systematically classified as follows: (i) epidemiological hygiene, or infection control, in terms of ensuring “epidemiological harmlessness,” which is achieved by hygiene measures, i.e., through pathogen elimination or inactivation; (ii) phytohygiene refers to the production and use of aseptic, germ-free substrates, which is achieved by treatment methods to kill pests and weed seeds introduced in particular by plant material (e.g., during composting); (iii) low pollution characteristics concern the prevention of excessive pollution of the environment (e.g., soil and water) with critical concentrations of substances such as heavy metals or microplastics, or organic trace substances such as pharmaceutical residues (drugs and hormones); (iv) horticultural suitability of the recycled products for use as fertilizer in horticulture concerns, for example, characteristics such as nutrient content, plant availability of nutrients (i.e., nutrient solubility), proof of fertilizer effect, and sufficient homogeneity and manageability of the product.

## Results

In this section, we discuss the risks related to the three substance groups defined above for use in horticulture, according to the identified four risk categories.

### Risks Associated with Human Excreta

Human feces, urine, blood, and vomit belong to the substance group of human excreta. The two key aspects considered when assessing their risk potential are epidemiological hygiene (“Epidemiological Hygiene of Human Excreta” section) and contamination with pharmaceutical residues as most relevant pollutant (“Pollution Characteristics of Human Excreta” section). Moreover, the horticultural suitability will also be discussed (“Horticultural Suitability of Human Excreta” section).

#### Epidemiological Hygiene of Human Excreta

The global spread of SARS-CoV-2 underscores the overall importance of public health and infection control. Even though the risk of transmission of SARS-CoV-2 from the fecal-oral pathway appears to be low [42, 43], there is a wide range of other pathogens found in human excreta. Hence, in the horticultural application of products recycled from human excrements, health risk plays a crucial role. Pathogens can belong to the categories of bacteria, viruses, protozoa, or helminths (gastrointestinal worms). According to Schönning and Stenström [16], most pathogens in human excreta are excreted with feces, as part of the large microbial content of feces: 25–54% of dry matter (DM) of feces consist of bacteria [44]. The eggs of helminths are also excreted with feces, with the exception of bilharzia, which is the only known worm whose eggs are known to be expelled via urine [16]. The World Health Organisation (WHO) has compiled an extensive list of pathogens excreted via human feces or transmitted via water and improper hygiene [19]. These pathogens are summarized in Table 1, and classified according to epidemiological risk: high, medium, low, or indeterminate.

**Table 1:** Pathogens excreted with human feces and their epidemiological relevance according to the WHO [19]

Category	Epidemiological risk			
	High	Medium	Low	Indeterminate
Bacteria	Enterohaemorrhagic *E. coli* Enteropathogenic *E. coli* *Salmonella enterica ser.* Typhi *Shigella dysenteriae* *Shigella flexneri* *Shigella sonnei* *Vibrio cholerae* AMR^a^ opportunistic pathogens	Enteroinvasive *E. coli* Enterotoxigenic *E. coli* *Yersinia enterocolitica*	*Campylobacter* spp. *Clostridium difficile* Other strains of *Salmonella*	Enteroagglomerative *E. coli* *Helicobacter pylori*
Viruses		Hepatitis A Hepatitis E Polioviruses	Adenoviruses Astroviruses Noroviruses Rotaviruses Sapoviruses	Enteroviruses
Protozoa	*Cryptosporidium* spp*.* *Entamoeba histolytica*	*Giardia intestinalis*	*Cyclospora cayetanensis*	
Helminths	*Ascaris lumbricoides* (roundworm) *Ancylostoma duodenale* *Necator americanus* (hookworm) *Hymenolepis* spp. (dwarf tapeworm) *Schistosoma haematobium* (blood fluke) Other *Schistosoma* spp. *Strongyloides stercoralis* (roundworm) *Taenia solium* (pork tapeworm) *Taenia saginata* (beef tapeworm) *Trichuris trichiura* (whipworm)	*Diphyllobothrium latum*		Trematodes (flatworms), parasites or flukes

^a^*AMR*, antimicrobial resistance

The hygiene risk of fresh human urine is low, as it is commonly assumed not to transport pathogens [45]. Urine is filtered in the kidneys and is sterile until it passes through the urinary tract, where it comes in contact with comparatively harmless germs (including lactobacilli). Few pathogens of human pathological relevance are excreted with urine [16, 19], and presented in Table 2. Of these, only *Schistosoma haematobium* poses a high epidemiological risk. The spread of this pathogen is usually endemic, and there is particular risk around sources of fresh water [19]. While fresh urine does not post a major hygienic risk due to the low content of pathogens, urine collected in urine-separating toilets can be more critical. Due to the cross-contamination with feces, it can contain considerable amounts of pathogens [16].

**Table 2:** Human urine-related pathogens and their epidemiological relevance [16, 19]

Category	Epidemiological relevance			
	High	Medium	Low	Indeterminate
Bacteria			Mycobacteria *Salmonella typhi* and *paratyphi* *Leptospira interrogans* Bacteria causing urinary tract infection^a^	
Viruses				Cytomegalovirus John Cunningham virus Humanes Polyomavirus Adenoviruses Hepatitis (probably low)
Fungi			Microsporidia	
Helminths	*Schistosoma haematobium*			

^a^Bacteria causing urinary tract infection such as. *E. coli*, *Enterococcus fecalis*, and others []

Contents from DTs also contain small amounts of blood—mainly menstrual blood, which can enter both the liquid and solid waste collection containers of DTs [46]. Many dangerous blood-borne pathogens exist, including hepatitis viruses, syphilis, malaria, and the human immunodeficiency virus (HIV) [47]. Pathogens that circulate in the blood during an infection can remain active in excreted blood. Transmission of blood-borne pathogens occurs through blood transfusion or other direct contact with blood. It has been postulated that menstrual blood does not pose any risk of disease transmission through DTs, or through the recycling of their contents [16]. However, we did not find any in-depth study on the fate of pathogens excreted with menstrual blood. Nevertheless, proper disposal management of menstrual hygiene articles is highly recommended, i.e., to collect these hygiene articles separately in waste bins placed in the DT nearby the toilet seat.

Finally, gastrointestinal infections, often accompanied by diarrhea and vomiting, are among the most common infectious diseases worldwide and can be transmitted not only through feces, but also via vomit [19]. However, the amounts of vomit collected in DTs is normally very low [16]. Noro- and rotaviruses are particularly relevant for children. The most important bacterial pathogens are *Salmonella*, *Campylobacter*, or *Escherichia coli* (e.g., the enterohemorrhagic strain) causing gastrointestinal infections that are usually very contagious.

In hygienization, a broad range of treatment methods exist with which pathogens can be killed or inactivated [15, 16]. The survival of microorganisms in the environment depends on many factors, including temperature, pH, water content, i.e., humidity, solar radiation, antagonists, and nutrient availability. Accordingly, hygienization can be achieved in a number of ways, including (i) heat treatment such as pasteurization [49, 50], thermophilic composting [28, 50, 51], or carbonization [31–33], and (ii) alkaline or acidic treatment with appropriate additives, e.g., urea or lactic acid bacteria [30] and (iii) drying [52]. Many treatment processes are discussed in the literature. A good overview is given by the German Association for Water Management, Sewage and Waste (*Deutsche Vereinigung für Wasserwirtschaft, Abwasser und Abfall*, DWA) [11] or Larsen et al. [14]. Heat treatment is arguably the most recommended process for sanitizing of solids, which contain feces. The WHO recommends a heat treatment at 55–60 °C over the course of several days with constant temperature monitoring [19]. For generic biowaste, the German Biowaste Ordinance (*Bioabfallverordnung*, BioAbfV) prescribes as treatment options (i) pasteurization of <70 °C for at least 1 h, (ii) thermophilic composting of either 55 °C for 2 weeks, 60 °C for 6 days, or 65 °C for 3 days, or (iii) thermophilic fermentation at <50 °C at an appropriate retention time for the specific digester [50]. In practice, the hygienization of contents from DTs is usually based on recommendation for sewage sludge treatment, which states that hygienization is to be achieved by pasteurization at a temperature of 70 to 80 °C for a short period (about 30 to 60 min) or by thermophilic composting with a hot decomposition phase and temperatures above 60 °C for several days [50, 53]. Also for urine treatment, pasteurization is used for sanitation, for example during the final distillation step [21]. However, the nitrogen compounds in urine need to be stabilized before heating; otherwise, N is lost by urea degradation [54] and ammonia (NH_3_) volatilization [55].

To sum up, when comparing the four excreta feces, urine, vomit, and menstrual blood, feces supposedly pose the highest risk, because it can contain a high number and large variety of pathogens and also contributes substantially to the mass of material in DT. The epidemiological risk of human vomit is in principle comparable to that of feces, but under normal conditions, only small amounts of vomit are collected in DTs. The risk associated with fresh urine is low, but it must be considered that in urine-separating toilets or even urinals, urine is usually cross-contaminated with other excreta. Menstrual blood or blood entering the DT due to injuries or with vomit make up a small amount of the total mass collected in the DT. It can therefore be assumed that the probability of contact with blood is low, when handling or treating excreta from DT. However, blood can contain dangerous and highly infectious pathogens. Based on our literature review, we cannot make a final assessment of the risk of pathogen transmission by blood. Finally, the presence of pathogens in human excreta does not generally impede their use as fertilizers, if they are treated appropriately. Actually, a wide range of treatment options exist. However, good care must be taken that also during collection and handling no transmission of pathogens occur. Considerable amount of research has been done on transmission pathways and recommendations for safe excreta handling exist such as the WHO Guidelines on Sanitation and Health [19].

#### Pollution Characteristics of Human Excreta

This section addresses the relevance of heavy metals, microplastics, and pharmaceutical residues as potential pollutants in human excreta.

Table 3 summarizes literature values for concentrations of heavy metals in human urine, feces, other waste streams, and recycling fertilizers. Please also see the “Horticultural Suitability of Human Excreta” section for the water or DM content in fresh matter (FM) for the various material flows. Compared with conventional biogenic waste streams and fertilizers, the heavy metal content in human feces is low. However, human intake of cadmium (Cd) is possible through food and tobacco consumption. Plants such as tobacco, soybeans, rice, and leafy vegetables can absorb Cd from the soil during cultivation [74]. In the case of tobacco in particular, Cd accumulates in the plant at concentrations far above soil concentrations [ibid.]. Drinking water pollution and direct exposure through the air generally contribute only to a small extent. Overall, comparing the potential risk for environmental pollution with heavy metals for a human urine-sourced fertilizer with the risk of a mineral P-fertilizers made from rock phosphate, the latter contain 3–20× more Cd, when referring to the same amount of nutrient application [4, 45]. With regard to the horticultural suitability of recycling fertilizers, it should be noted that certain heavy metals in low concentrations, e.g., copper (Cu), zinc (Zn), nickel (Ni), iron, manganese, molybdenum, and cobalt, are essential for plant physiology as micronutrient elements [75].

**Table 3:** Heavy metal content in human urine, feces, and other waste streams or recycling fertilizers. The values are given as mean ± standard deviation of a total of n values determined from the data in the sources. For urine, values are given in μg L^−1^ of FM, for all other material flows in mg kg^-1^ of DM. Where source FM values related were converted to DM, Gaussian error propagation was applied

Material flow	Cr	Cd	Cu	Ni	Pb	Zn
	μg L^−1^ FM for urine; mg kg^−1^ DM for all other material flows					
Urine [11, 58–60]	<5 or 7.1 (n=2 or 1)	<0.2 or 0.7 (n=2 or 1)	<10 or 71.4 (n=2 or 1)	<10 (n=3)	<0.8 or 14 (n=3 or 1)	194±88 (n=3)
Feces [44, 59]	1.8±1.2 (n=3)	0.7±0.6 (n=2)	26.9±9.7 (n=2)	4.2±2.0 (n=3)	0.7±0.3 (n=3)	241±90 (n=3)
Sewage sludge [61, 62]	28.0±12.7 (n=2)	0.8±0.2 (n=2)	366±93 (n=2)	20±7 (n=2)	27±14 (n=2)	658±80 (n=2)
Cattle slurry [63, 64]	2.4±2.1 (n=3)	0.25±0.05 (n=2)	35.3±12.4 (n=4)	5.4 (n=1)	2.8±1.4 (n=4)	138±58 (n=4)
Pig manure [63, 64]	3.1±2.7 (n=4)	0.20±0.13 (n=3)	612±362 (n=4)	9.8 (n=1)	3.0±1.0 (n=4)	702±158 (n=4)
Organic waste compost [64–66]	24.9±3.3 (n=4)	0.5±0.2 (n=4)	52.4±12.6 (n=4)	15.8±3.5 (n=4)	40.1±12.5 (n=4)	204±54 (n=4)
Gardening waste compost [66–70]	29.5±28.1 (n=5)	0.32±0.06 (n=5)	36.4±27.2 (n=5)	16.5±10.2 (n=5)	24.1±5.6 (n=4)	161±116 (n=5)
Digestate compost [71–73]	28.7±27.1 (n=8)	0.8±0.6 (n=8)	50.3±14.1 (n=8)	17.3±13.0 (n=8)	53.5±45.4 (n=8)	251±66 (n=8)

*Cd*, cadmium; *Cr*, chromium; *Cu*, copper; *DM*, dry matter; *FM*, fresh matter; *Ni*, nickel; *Pb*, lead; *Zn*, zinc

Since microplastics were recently reported in human feces, with 20 such particles of 50–500-μm diameter detected in 10 g feces [74, 75], these should also be considered a potential pollutant in human excreta. By comparison, 14–895 microplastic particles of up to 1–5-mm diameter per kilogram of DM and an unspecified amount of smaller particles were found in composts from biowaste [76]. It remains unclear whether microplastics pose a problem in this context, as they occur in different places in the environment, particularly the hydrosphere, and are not a problem specific to recycling fertilizers produced from DT contents.

Pharmaceutical residues, such as drug residues and hormones, represent another risk in terms of harmful substances. Overall, to the best of our knowledge, little is known about the distribution or concentrations of pharmaceuticals in the environment, particularly in soil, or about the human- and eco-toxicological effects of these residues or their degradation or metabolic (by)products (metabolites). Previous studies have focused mainly on the levels and degradation of pharmaceuticals in wastewater and running water, e.g., [77–81]. The scientific state of knowledge regarding the pharmaceutical risk of urine and feces is currently being developed. However, soil is generally better suited than water for the degradation of pharmaceutical residues [19]. Moreover, new pharmaceutical substances are constantly being introduced to the market. In the following, we summarize our findings on the relevance of pharmaceutical residues in urine and feces.

According to the German Federal Environment Agency (*Umweltbundesamt,* UBA), the consumption of pharmaceuticals in Germany increased from ~5,500 tons to almost 7,100 tons between 2002 and 2009 [82]. This corresponds to an increase of ~30%, whereas the use of certain individual substances, such as the hormones norgestimate or dydrogesterone, demonstrated increases of several to 1,000% within 6 years [83]. The pharmaceuticals also end up in the environment. Median active substance concentrations of pharmaceuticals in general are typically >0.5 μg L^−1^ in surface waters and >0.1 μg L^−1^ in groundwater, which is in close connection to surface water [ibid.]. The EU guideline for the environmental assessment of medical products for human use specifies a threshold value of 0.01 μg L^−1^ [84]. The concentrations found in waterbodies thus exceed this threshold and, therefore, pose a potential risk to the environment and human health [85].

Pharmaceutical residues enter DT contents primarily via urine and feces, with different chemicals excreted via each waste stream. They may also be present in blood and vomit [86–88], but their share to DT content is marginal compared to human excreta. For this reason, we will only look into the relevance of residues in urine and feces. Most (~64%) drugs are excreted with urine after they or their metabolites are processed via the kidneys, and about one-third is eliminated with feces [89]. The distribution of substances in the two waste streams varies according to their chemical properties, but the associated risk potential is similar [90, 91]. Overall, according to our research, there are currently significantly fewer literature entries and data on the deposition of pharmaceutical substances and their metabolites via feces than via urine.

Substances that are predominantly excreted with feces [92] include lipophilic substances (e.g., naproxen, diclofenac) that tend to bioaccumulate and generally pose a higher eco-toxicological risk than hydrophilic substances [90, 91, 93]. The risk also depends on whether the substances can spread in the environment. The main residues excreted with urine are non-metabolized active substances and conjugates that are susceptible to subsequent transformation in the environment by hydrolysis, as well as metabolites, of which some are highly bioactive [88]. Therefore, when assessing the risk of pharmaceuticals, non-metabolized active substances and metabolites must be considered. Furthermore, it must also be considered that conjugated substances can be hydrolyzed to their original active form. Urine contains mainly hydrophilic active substances (e.g., acetaminophen, sulfamethoxazole) [92], which, although they do not bioaccumulate to the extent that hydrophobic substances do, dissolve more easily in water and are distributed in this way, enter water bodies, or are taken up by plant roots [89, 94].

Several processes have been investigated for their effectiveness to eliminate pharmaceuticals, especially for urine. Özel-Duygan et al. [95] showed that a small share of pharmaceuticals is removed during storage, some more during biological treatment, i.e., nitrification. Extensive removal below the limit of detection can be achieved with powered [95] and granulated activated carbon [96]. Further processes for pharmaceutical removal from urine are discussed in [95, 96]. The elimination of pharmaceuticals during thermal composting of DT contents is currently being studied, with promising indications [97, 98]. Targeted treatment of urine and feces collected in DTs will not only improve the quality of the derived fertilizers, but it will also be a more efficient way to prevent environmental pollution by pharmaceuticals, because the treatment can be more effective than in centralized wastewater treatment plants [96].

In order to assess the risk of pharmaceuticals associated with applying DT residues for horticultural applications, a comparison with conventional recycling products such as farmyard manure and sewage sludge can be considered. Table 4 lists the range of concentrations of residues detected in human urine, sewage sludge, various P-recycling fertilizers from sewage sludge, animal manure, and soil. Our search did not reveal any data for pharmaceutical residues in human feces; data for separately collected urine (e.g., from separation toilets) were significantly less than for sewage sludge. The table further contains a selection of the substances investigated with largest data overlap in the different matrices, and not the complete pharmaceutical content of the materials. Moreover, most studies only focus on the parent pharmaceutical, and do not consider their metabolized conjugates. This is particularly relevant for hormones (e.g., natural and synthetic estrogens), as they are mainly excreted as conjugated form (e.g., estrone-sulfate, estradiol-sulfate), which can be deconjugated (e.g., by *E. coli*) to the active compound [107]. Hence, the total hormone load in the matrices might be underestimated by only analyzing the parent drug. This is an analytical issue that applies to other substances as well [108]. Brown and Wong [ibid.] stressed the importance of also analyzing phase II conjugates to evaluate the total compound load of pharmaceutical substances. In summary, Table 4 shows that pharmaceutical loads in urine vary widely, which depends on whether the sample is from family homes, residential complexes, or public urinals. Maximum pharmaceutical levels in urine are generally significantly lower than those in sewage sludge. The compounds found in high concentrations in soil are similar to those in manure, e.g., the antibiotics enrofloxacin, tetracycline, and chlortetracycline. P-recyclates from sewage sludge may still contain pharmaceutical residues, depending on the production process; however, in products of thermal treatment processes with more than 210 °C (e.g., P-ashes, biochars, and hydrochars: Ash-Dec®, PYREG®, TCR®30), no substances were detected [109]. In the production of magnesium ammonium phosphate (MAP), ultrafiltration can shift the pharmaceutical contents towards the minimum values seen in Table 4 [ibid.].

**Table 4:** Ranges of concentrations of pharmaceutical residues in different substrates, where (n) depicts the number of data [11, 82, 99–104]

Pharmaceutical	Urine^a^			Liquid manure^b^	Sewage sludge^b^	P-ROC^a,c^			MAP			Soil
	μg L^−1^			μg kg^−1^	μg kg^−1^	μg kg^−1^			μg kg^−1^			μg kg^−1^
	Min	Max	Mean (n)	Max (n)	Max (n)	Min	Max	Mean (n)	Min	Max	Mean (n)	Max (n)
17-α-Ethinylestradiol					313 (28)	0.2	2.5	1.4 (4)	2.5	25	5 (9)	67.3 (4)
17-ß-Estradiol	<50		- (1)	392 (1)	836 (20)	0.2	7.3	3.4 (4)	2.5	25	5 (9)	26.9 (3)
Bezafibrate	163	573	364 (3)		640 (16)	0.27	2.5	1 (4)	0.5	8.4	2.6 (9)	
Carbamazepine	0.25	124	22.8 (5)		680 (57)	1.2	2.9	2 (4)	0.5	230	67.3 (9)	1.5 (5)
Chlortetracycline				203 300 (13)	107 (1)							820 (40)
Ciprofloxacin^d^		13	13 (1)	28 (10)	41 800 (40)	2	270	106 (4)	4.8	1 100	263 (9)	4.6 (2)
Clarithromycin	<1	300	17 (1)		180 (15)	0.23	14	6.7 (4)	2.5	50	9.6 (9)	
Diclofenac	0.25	56	18 (7)		627 (50)	2.9	8.7	5.2 (4)	0.5	38	13.4 (9)	0.1 (2)
Enrofloxacin^d^				8 300 (17)	20.7 (1)							3 810 (3)
Fenofibrate					302 (13)							
Ibuprofen	13	4 160	957 (7)		3 237 (73)							16.3 (7)
Ketoprofen	<1	13.6	13.6 (3)		131 (16)							97.3 (5)
Metoprolol					21 100 (10)	0.27	200	64.4 (4)	0.5	420	112 (9)	0.3 (6)
Naproxen	7.9	386	146 (4)		5 460 (55)							0.2 (2)
Estrone	1.1	7.5	3.4 (4)	1 068 (2)	887 (14)							62.2 (7)
Paracetamol	36	140	88 (2)		419 (35)							1.8 (2)
Phenazone	2	4	3 (2)		36.7 (3)							
Progesterone	1.6	52	26.8 (2)		273 (1)							
Sulfadiazine				91 000 (9)	112 (5)							60.3 (12)
Sulfadimidine				167 000 (21)								16 800 (15)
Sulfamethoxazole	<2	6 800	14.2 (1)	20 (1)	178 (32)	0.05	5	1.5 (4)	0.5	10	2.5 (9)	239 (12)
Tetracycline	36.2	2 300	1 168 (2)	66 000 (24)	37.2 (1)							395 (31)
Trimethoprim	0.06	1 300	64 (3)	17 000 (4)	188 (23)							100 (3)

^a^Data retrieved from literature search. ^b^Data retrieved from the UBA database “Pharmaceuticals in the environment” [105] with search terms provided in Appendix A2. The individual values taken from the database have different units: μg L^−1^, μg kg^−1^ DM, μg kg^−1^ FM, or μg kg^−1^ without specifying DM or FM (see also Appendix A2). To allow comparisons and to take into account the majority of values for which DM or FM is not specified, the values were listed in μg kg^−1^ or μg L^−1^. To compare μg L^−1^ with μg kg^−1^, we assumed 1 L urine corresponds to 1 kg material. We further assumed that the maximum values in sewage sludge, manure, and soil are related to kg DM. ^c^Crystallization product of sewage sludge that may contain Ca_3_(PO_4_)_2_ and MAP [106]. *DM* dry matter, FM fresh matter, *MAP* magnesium ammonium phosphate, *P-ROC* P-recycling products from sewage sludge. ^d^The human antibiotic ciprofloxacin is a degradation product of the veterinary drug enrofloxacin, which accumulates in the soil on application of liquid manure [87]

The environmental risk of drug residues is assessed primarily from their eco-toxicological effect in receiving waters, their behavior in soil, and their uptake by organisms and plants [110, 111]. Winker et al. modeled (based on the work of Hammer and Clemens [112]) amounts of antibiotics applied per hectare per year under optimal fertilizer application to meet nutrient requirements with pig and cattle slurry compared with human urine from German volunteers [4]. They found that fluxes of antibiotics applied with human urine would be at least 100 times lower compared to the input with animal manure [ibid.]. Hormones show a trend pointing in the same direction, however, with the exception of 17a-ethinylestradiol, which would be applied in a higher amount with human urine than with cattle manure. Bischel et al. [113] estimated the eco-toxicological risk for soils, if urine from urine diverting dry toilets were used as fertilizers in Durban/South Africa. To quantify the risk, they compared the risk quotient (RQ) of the predicted environmental concentrations (PEC) over the predicted no effect concentrations in soils (PNEC). They found that RQ was far below 1 for trimethoprim and diclofenac, which means that the risk is low. However, RQ for the antibiotic sulfamethoxazole was above 1, showing that there is a potentially high risk. The approach of calculating RQs for risk assessment has been widely used for aquatic ecosystems, see, e.g., [114]. For soil ecosystems, this approach is challenged by the low availability of PNEC for soils and the unknown effect of urine and water application rates, soil characteristics, and pharmaceutical degradation rates. Furthermore, Konradi et al. [110] developed an evaluation method for systematic classification and assessment of the potential environmental hazard of human pharmaceutical residues in sewage sludge for soil and soil organisms (Appendix A1). The classification of the hazard potential and the associated prioritization of pharmaceuticals result from the sum of the classifications for sewage treatment plants, soil, and eco-toxicological effects based on existing literature. The following pose a particular risk for soil and are recommended as indicators for risk assessment or quality assurance: (i) antibiotics ofloxacin, ciprofloxacin, norfloxacin, roxithromycin; (ii) antiepileptic drug carbamazepine; (iii) hormones ethinylestradiol, estradiol; and (iv) lipid-modifying agent fenofibrate [110].

Since the analysis of pharmaceuticals and hormones is material-, time-, and cost-intensive, it is therefore practically impossible to perform laboratory analysis for all pharmaceutical substances available on the market. Hence, screening the recycled products for the recommended indicator substances is of high practical relevance to assess possible negative environmental effects of drug residues on DT content–derived fertilizers.

Ultimately, the uptake of active substances via plant roots and eventually shoots, leaves, or fruits is possible, but also depends on the drug’s chemical properties and on environmental factors [115]. Arnold and Schmidt [116] demonstrated that diclofenac, atenolol, and verapamil are not transferred from urine-based fertilizer to crops. Furthermore, carbamazepine was found in wheat and maize grains and stems, but in relatively low concentrations [ibid.]. Considering the average amount of grain consumed per year by a person in Germany (~100 kg), one would need to eat wheat for >100 years to ingest the daily dose of carbamazepine given to a person with epilepsy (>400 mg). An assessment of the potential for translocation of the drugs carbamazepine, sulfamethoxazole, and triclosan into the edible part of the plant was investigated for different species. The results indicated that the potential for uptake decreases in the following order: leafy vegetables > root vegetables > cereals and fruit-bearing vegetables [115]. The daily human consumption of contaminated vegetables needed to reach toxic limits is not considered realistic [115, 117]. Many studies have also investigated the uptake of various active pharmaceutical substances contained in wastewater, sewage sludge, urine, and animal manure (e.g., carbamazepine, diclofenac, ibuprofen, salbutamol, sulfamethoxazole, and trimethoprim) into the plant [94, 117–124].

#### Horticultural Suitability of Human Excreta

The specific horticultural application of fertilizer products originating from human excreta or DT contents mostly depends on their macronutrient content and nutrient ratios. Other relevant properties include the salt content, water content, and physical structure.

Most relevant plant micro- and macronutrients can be found in urine and feces (Table 5). The nutrient concentration in urine and feces can vary depending on diet and correlates, for example, with the protein content in the food consumed [135]. Similarly, the nutrient content in liquid manure, compost, or digestate can also fluctuate, as it is highly dependent on the type and quantity of input material [136]. To estimate the N and P content in human urine and feces, formulae have been developed based on “national food balances” reported by the United Nations Food and Agriculture Organisation [135]. This approach has been validated for several countries, including China, Germany, South Africa, Sweden, and Ethiopia [125, 135].

**Table 5:** Nutrient contents in human urine, feces, and other waste streams or recycling fertilizers. The values correspond to the mean ± standard deviation determined from the data in the sources. For urine, values are given in g L^−1^ of FM; for all other material flows in g kg^−1^ of DM. Where source FM values were converted to DM, Gaussian error propagation was applied

Material flow	N	NH_4_^+^-N	P	K	Mg	Ca
	g L^-1^ FM for urine, or g kg^-1^ DM for all other material flows					
Urine [4, 8, 11, 44, 58, 60, 125, 126]	7.1±3.5 (n=22)	0.58±0.49 (n=6)	0.8±0.6 (n=15)	1.5±0.7 (n=14)	0.06±0.05 (n=6)	0.10±0.09 (n=6)
Feces [8, 44, 126, 127]	28.2±8.0 (n=8)	n.d.	7.8±2.9 (n=14)	10.3±4.0 (n=15)	3.4±2.3 (n=5)	17.7±9.4 (n=7)
Sewage sludge [62, 64, 69]	48.7±8.5 (n=3)	11 (n=1)	26.4±5.7 (n=3)	3.0±0.6 (n=2)	4.3 (n=1)	38.6 (n=1)
Cattle slurry [11, 64, 128]	43.8±5.3 (n=2)	25.6 (n=1)	7.9±1.2 (n=2)	48.7±4.3 (n=2)	4.8 (n=1)	12.8 (n=1)
Pig manure [11, 64, 128]	82.8±15.2 (n=2)	62.5 (n=1)	23.2±1.6 (n=2)	43.7±10.1 (n=2)	8.4 (n=1)	12.0 (n=1)
Bio-waste compost [64, 66, 127–129]	10.0±7.9 (n=5)	1.1±0.3 (n=2)	3.0±2.5 (n=6)	7.9±5.5 (n=6)	5.8±8.3 (n=4)	90 (n=1)
Garden waste compost [26, 66, 68, 70, 128, 130, 131]	10.7±2.5 (n=7)	0.22±0.15 (n=2)	2.3±0.8 (n=7)	8.0±1.9 (n=7)	5.3±2.3 (n=6)	25.3±12.3 (n=4)
Digestate compost [72, 73, 132–134]	38.0±15.0 (n=9)	2.8±2.8 (n=9)	10.0±5.5 (n=9)	19.2±9.4 (n=9)	4.1±1.4 (n=7)	43.7±18.6 (n=7)

*DM* dry matter, *FM* fresh matter, *n.d.* no data

Humans excrete nutrients mostly via the urine: ~90% of N, 50 to 65% of P, and 50 to 80% of K [58, 59, 137]. This distribution is reflected in the ratio of the nutrients N, P, and K in urine and feces; the NPK ratio in urine is ~18:2:5, and in feces DM it is ~ 3:1:1 [138–140]. Urine is therefore rich in N and feces rich in P. Urine is further characterized by a balanced N to K ratio, matching the nutrient requirements of many crops; however, its P content is quite low. Urine-based fertilizers are therefore well suited for soils with P surpluses, such as those found in Germany. By comparison, digestate and pig or cattle slurry have a much narrower N to P ratio, risking over-fertilization with P when trying to provide sufficient N; accordingly, with sufficient P fertilization, additional N is required. Soils with a low P content can benefit from feces-based fertilizers, which are good P suppliers, similar to digestate and liquid manure. Most of the calcium (Ca) and magnesium (Mg) excreted by humans are contained in the feces.

Besides the total amount of nutrients in fertilizers, their availability for plants is a critical characteristic, determined by factors such as water solubility, as the mineral forms of N in fertilizers—ammonium (NH_4_^+^) and nitrate (NO_3_^−^)—are water-soluble and thus potentially directly available to plants. Recycling fertilizers with a high proportion of mineral N (N_min_) generally have a good *short-term* fertilizing effect. However, the ionic N forms differ in mobility within the soil solution: NH_4_^+^ can easily bind to the negatively charged clay particles and organic matter (fixation), or can be consumed by microorganisms (immobilization) [141], whereas NO_3_^−^ is highly mobile and prone to leaching [142]. In fresh urine, N is present as urea (CH_4_N_2_O), which is hydrolyzed to NH_3_ by the enzyme urease. Hence, N in stored urine is present mainly as the non-ionic form free NH_3_ or NH_4_^+^ (because NH_4_^+^ and NH_3_ are in equilibrium). A recently developed treatment process to optimize the form of nutrients in urine is nitrification, whereby NH_3_, respectively NH_4_^+^, is biologically oxidized to NO_3_^−^ [21, 22, 143]. By adding a base (increasing pH) during the nitrification process, the ratio between NH_4_^+^ and NO_3_^−^ can be adjusted for different types of plant production [21, 22, 143, 144]. Animal manure or fresh digestate from biogas production also contain relatively high NH_4_^+^ concentrations (Table 5) [145]. However, potential negative impacts on seed germination and seedlings have been reported for application of biogas digestate, which were related to phytotoxicity by NH_4_^+^/NH_3_, often referred to as “NH_4_^+^ toxicity” [146, 147], organic acids, and the increase of soil electrical conductivity [145, 148–150]. This NH_4_^+^ toxicity could also occur during fertilization with untreated or stored urine, if applied directly after sowing or on young plants. For biogas digestate, it was suggested that the risk of these negative effects will decrease shortly after application to the soil [145], which would probably also apply for stored urine. It should be noted that some urine treatment processes, such as acidification by acid dosage or nitrification, lower the content of free NH_3_ considerably (i.e., by shifting the equilibrium NH_4_^+^ + OH^−^ ⇌ NH_3_+H_2_O) and therefore reduce the negative effect of NH_4_^+^ toxicity [55]. Feces contain a significant proportion of organically bound N (N_org_), and are therefore more likely to be characterized by a *long-term* fertilizing effect. For DT content–derived compost, the availability of the nutrients to plants is similar to that in composts derived from raw materials of plant or animal origin. The nutrients are bound mainly in the organic matrix and can be mineralized. Fresh urine has a very low organic C to N ratio of <1 (or 0.4 not considering C in urea [151]). During the production of a urine fertilizer via nitrification, the C content is reduced even by 90% [55]. Urine and urine-derived fertilizers are therefore N fertilizers with high nutrient available as urea, NH_4_^+^ or NO_3_^−^ [152]. In contrast, feces have a significantly higher C to N ratio, contributing to humus reproduction, C binding, and C storage in the soil [153].

Empirically, a positive fertilizing effect has been demonstrated for both urine and feces; this applies to the nutrient content and the plant availability. Urine-based fertilizers demonstrate nutrient plant availability comparable to mineral fertilizers containing urea, NH_4_^+^ or NO_3_^−^ [4, 59, 154, 155] or phosphate (PO_4_^3−^) [116, 156]. Due to its good fertilizing properties, urine is suitable as a substitute for synthetic fertilizers in the cultivation of crops such as maize, beans, wheat, barley, or miscanthus [52, 58, 149, 155, 157–159]. A long-term experiment with 11 years of repeated urine application revealed that the mineral fertilizer equivalent, hence the proportion of N use efficiency compared to mineral NPK fertilizer, can reach 82% [152]. Moreover, the potential positive effects of adding urine to compost to increase the N content and regulate the water content during composting have been discussed previously as recommendation to practice [160]. By contrast, feces-based fertilizers such as compost exhibit a slower nutrient availability for plants [59]. However, this material has demonstrated positive effects on soil organic matter and thus, improvement of soil structure and increase of water holding capacity and buffer capacity [ibid.] as well as soil pH [149].

Besides their nutrient content, the physical structure of the fertilizer product is also relevant for practical applications. For example, it determines the spreading and storage life of the recycled product. A vital feature of recycling fertilizers in this context is their water content (Table 6). Liquid materials can be pumped and can be spread using techniques that are already established from fertilizing with liquid manure [165, 166]. On the other hand, for the application of solid fertilizers, spreaders for mineral fertilizers, manure, and compost are suitable [167]. In general, following application, a superficial incorporation (e.g., with a cultivator, disc harrow) should be carried out immediately to improve biological conversion and nutrient availability [153, 168].

**Table 6:** Proportion of DM in FM for human urine, feces, and other waste streams or recycling fertilizers. The values correspond to the mean ± standard deviation determined from the data in the sources

Material flow	% DM in FM
Urine [4, 59, 126]	3.1±1.2 (n=4)
Feces [44, 126]	25±0 (n=2)
Sewage sludge [64]	3.5 to 25 (n=1)
Cattle slurry [64, 128]	9.8±3.2 (n=2)
Pig manure [64, 128]	5±0 (n=2)
Organic waste compost [64, 66, 127–129]	62.8±2.0 (n=5)
Gardening waste compost [26, 66, 68, 70, 128–131]	53.3±11.7 (n=7)
Digestate compost [73, 132–134]	46.4±21.3 (n=8)
Food leftovers [63, 161–164]	34.4±7.2 (n=13)

*DM* dry matter, *FM* fresh matter

There are also possible horticultural risks associated with recycling fertilizers from human excreta. Depending on the diet, urine often has an increased salt content in the form of sodium chloride (NaCl), which can increase the electrical conductivity of soils [169]. Based on the amount or frequency of application, excess NaCl may affect salt-sensitive crops or have a negative impact on soil structure [170–172]. However, no negative impacts on soil fertility and plant growth, related to salt build-up in the soil, could be detected after 11 years of continuous urine application in Denmark [152]. Salinization affects mainly soils in arid and semi-arid areas with low precipitation, or where no leaching occurs [59, 173]. However, further research is required to fully assess this risk. The impact of soil and groundwater salinization should also be compared with that of other, conventional fertilizers. For example, the production of conventional K fertilizers is associated with high salt input to the environment and accompanying negative effects like pollution of receiving waters [174].

Further negative environmental consequences can result from the emission of climate-impacting greenhouse gases (GHGs), such as nitrous oxide (N_2_O) or methane (CH_4_). Gaseous NH_3_ emissions also contribute to eutrophication. In the air, NH_3_ gas reacts with sulfuric and nitric acids to form salts that can easily be transferred to the pedosphere or hydrosphere with precipitation. These salts are readily water-soluble, leading to nutrient accumulation in the water and consequently to excessive growth of plants and algae (“eutrophication”). As mentioned above, stored urine, which was not stabilized, contains N_min_ predominantly as NH_4_^+^ or NH_3_. Depending on pH and temperature, the equilibrium can shift to gaseous NH_3_, which can volatilize. High pH, low buffer capacity, low soil moisture, high temperatures, and wind favor NH_3_ losses after fertilization [106] with harmful environmental effects of NH_3_ [175]. The following conditions at application will help avoid GHG or NH_3_ emissions: (i) cool-to-moderate temperatures; (ii) dry (not water-saturated) soil; (iii) incorporation of the fertilizer into the soil within 4 h of application, with the exception of compost and fertilizers with a DM content of <2% of FM [176]; (iv) low-emission application techniques such as drag hoses, drag shoes or injection; and (v) prior acidification of the fertilizer [52, 106, 145, 177–181]. To reduce N_2_O emissions, nitrification inhibitors [181, 182] and the aforementioned appropriate application techniques are suitable. In a recent field study, for instance, application with a drag hose and direct incorporation resulted in lower GHG release, mainly due to a reduction in direct N_2_O emissions [182]. The above recommendations correspond to the state-of-the-art in science and technology for fertilization with liquid manure or digestate, and can be used similarly for fertilizing with untreated, stored, or treated urine. When using recycling fertilizers in hydroponics, e.g., tomato production, N_2_O emissions of nitrified urine can be significantly lower compared to other recycling fertilizers, such as struvite and vinasse, which is due to the low organic-C content in urine [183]. Finally, the direct application of untreated or only stored urine can lead to unpleasant odors [184]. In practice, this is probably the most important obstacle to direct application. It should be noted that NH_3_ loss as well as malodors during fertilization can be prevented by treating stored urine with the nitrification process [55].

### Risks Associated with Operational Additives

In many cases, recycled products from DTs do not consist only of human excreta. Common additives therefore include toilet-related materials such as toilet paper, bedding material or detergents, and recovery additives for composting or urine treatment. Not all additive groups pose risks. In the following, the risk potential of additives is discussed, focusing on (i) the hygiene-related risk of toilet paper, (ii) phytosanitary hazards of treatment-related additives, (iii) pollutants, and (iv) horticultural suitability.

#### Epidemiological Hygiene of Operational Additives

Additives such as toilet paper do not *per se* pose a health hazard to humans. However, through contact with human excreta, they can become contaminated with pathogens from feces, urine, blood, or vomit. If toilet paper is not collected and disposed of separately, it becomes part of parent raw materials for fertilizer production, which needs to be treated for sanitization and tested for hygienic parameters.

#### Phytohygiene Relevance of Operational Additives

Phytohygiene deals with eliminating plant pathogens or weed seeds. This is relevant primarily for recycling fertilizers, which are produced with plant additives. One prominent example is compost. The addition of materials such as kitchen waste, green waste, straw, or other harvest residues to the DT contents is advisable for composting, as this optimizes the decomposition process and the quality of the resulting fertilizer [185] (see also “Horticultural Suitability of Operational Additives” section). However, composts that contain plant material including germinable seeds and sections capable of sprouting (e.g., garden and park waste) introduce the risk of weed invasion when applied as fertilizer. Particularly resistant seeds are those of tomato, which can be found in green waste from allotments and kitchen waste [186, 187]. The Federal Compost Quality Association (*Bundesgütegemeinschaft Kompost*, BGK) summarizes the requirements for the elimination of pathogens or weed seeds as follows: “The decisive factor for the killing of pathogens or weed seeds in composting is primarily the heat effect in the decomposition process. High temperatures over a long period of time, in combination with the corresponding humidity, kill pathogens. In addition, microbial activity in the rotting process, antagonistic effects or toxic degradation products also play a role in killing pathogens” [188]. A list of lethal temperatures for various pathogens, pests, and weed seeds has been published by the BGK [188]. In compliance with the requirements of the BioAbfV for commercial composting, an effective heat treatment a temperature of ≥55 °C should be maintained over a period of several days to weeks (cf. “Epidemiological Hygiene of Human Excreta” section) to kill pathogens and to inactivate the germination and sprouting capacity of seeds and weeds [50]. Finally, the compost must be tested for the effectiveness of sanitization. The BioAbfV stipulates a maximum of two plant parts able to sprout or germinable seeds per liter of test substrate [50]. For “small-scale” composting, like it is realized in household and kitchen gardens or allotments, reaching temperatures of ≥55 °C may not be possible. Composting on a small scale (<5 m^3^ year^−1^) usually takes place in the mesophilic range (<45 °C) that does not guarantee phytohygiene. Parts of plants that have been infested with diseases should thus not be placed in the garden compost, but should be discarded in household waste for incineration.

#### Pollution Characteristics of Operational Additives

Regarding the risks of pollutants in operational additives, heavy metals or chemical additives with negative environmental effects play an important role. This section discusses the risks of pollutants found in toilet-related additives (toilet paper, bedding material, detergents) and process-related recovery additives used for composting or the production of urine-based fertilizers.

With regard to toilet-related additives, conventional cellulose-based toilet paper is not problematic in its biodegradability. However, chemicals can be present in “tissue paper,” of which toilet paper often consists, including (i) chemical additives used in the production of soft multi-ply toilet paper, (ii) bleaching agents, (iii) printing ink or ink residues in recycled toilet paper, and (iv) additives in moist paper. Possible pollutants added at various stages of the production process are thereby wet strength agents, retention agents, defoamers, complexing agents, dyes, dispersants, fixing agents, and surfactants [175]. Wet-strengthening agents (e.g., urea-formaldehyde resins, melamine-formaldehyde resins, or polyacrylamide glyoxal) increase tear resistance when the paper is moistened. However, high levels of these agents would be detrimental to the wastewater treatment process so they are added only in small quantities [189]. Retention agents, also known as flocculants, including inorganic compounds (e.g., aluminum sulfate and polyaluminium chloride), modified natural materials (e.g., modified starch, galactomannans and carboxylmethyl cellulose), and synthetic-organic polymers (e.g., polyacrylamides and polyethylenimine), which are used either individually or in combination, are not known to impact the environment negatively at the concentrations used in the toilet paper production [190]. Numerous different agents are further used for defoaming (e.g., silicone oils, hydrocarbons and alkylene oxide adducts), deaeration (e.g., aliphatic alcohols, fatty acid esters and fatty alcohols) or combatting pollutants (e.g., short chain polymers and minerals) in the production process of tissue paper [189]. We cannot go into further detail about the risks of all these chemical additives as they mainly concern the papermaking process itself and the waste water and its treatment [191, 192]. However, in the following, the most relevant chemicals contained in tissue paper are addressed.

Chlorine (Cl)-bleached toilet paper, as another example of toilet-related additives, is a substantial source of adsorbable organic halogens (AOXs) [193]. However, paper manufacturing processes have significantly improved in recent decades. For example, the bleaching of recovered paper was developed as an elemental-Cl-free process about 25 years ago, largely replacing the use of hypochlorite as a bleaching agent. Nowadays, pulp bleaching is performed mainly using oxygen, hydrogen peroxide, and ozone, resulting in products with significantly lower AOX concentrations [194]. Significant improvements have also been achieved recently in the de-inking process, during which printing inks on wastepaper are removed, enabling substantial reduction of heavy metals from printing inks introduced in toilet paper [194]. Subsequent washing ensures further reduction of carbonates. The German Federal Institute for Risk Assessment [195] specifies limits for formaldehyde, glyoxal, and polychlorinated biphenyls (PCBs) in the quality assessment of sanitary paper. However, *Stiftung Warentest* [196] tested 27 types of toilet papers purchased in Germany for heavy metals, formaldehyde, and PCB, of which none were detected. Furthermore, a study by the Danish Environmental Protection Agency demonstrated that toilet paper made entirely or partly from recycled paper does not contain higher levels of organic chemical compounds, heavy metals, or other potentially harmful substances than the non-recycled kind, but toilet paper with printing, perfuming, or embalming contained significantly higher levels of these potentially harmful compounds [197]. However, bisphenol A (BPA) has been found in recycled papers (e.g., kitchen tissue, toilet paper), caused by thermal paper (e.g., receipts) containing high BPA concentrations and entering the recycling process [198–200]. BPA concentrations in toilet paper were found in a range of 0.0018–0.180 μg g^−1^ and evaluated to be of minor importance compared to dietary exposure [198]. Moreover, BPA has been shown to be biodegradable under aerobic conditions, e.g., during composting [201, 202]. In countries where the aforementioned optimizations are not applied in pulp processing and paper recycling, relevant amounts of pollutants such as Pb and Cd may still be present in recycled toilet paper [203].

Furthermore, the increasing use of wet wipes, as a substitute for toilet paper, represents a major challenge for processing DT contents. Polyester fabrics are not biodegradable, contributing to microplastic pollution [204], and are by far the most common foreign material in readily composted solids from public DTs. Additionally, they contain high concentrations of disinfectants, fragrances, and other chemical additives (see below for pollutants in detergents). Wet wipes are also a problem in water-based sanitation systems: when flushed down in the toilet, they get tangled together inside sewer channels, creating large obstructions [205].

During collection of human excreta from DTs, additives are used in most toilet systems, especially in composting toilets. These additives include bedding materials, i.e., “dry flush replacement,” added to the feces after each toilet use to visibly cover the droppings and to bind moisture and odors. These materials can consist of sawdust, wood shavings, powdery charcoal referred to as “biochar” (cf. “Horticultural Suitability of Operational Additives” section), rock flour, ashes, compost, or a mixture of these. Sawdust and wood shavings from the processing of natural wood thereby pose a low risk of pollutants. This is not the case for chips from the processing of glued and paneled wood and wood composites, which are increasingly used in modern joinery and the timber industry. Macroscopically, these hardly differ from natural wood waste, but can contain a variety of contaminants, such as alkanes, alkenes, aromatics, terpenes, halogenated hydrocarbons, esters, aldehydes, and ketones [206].

In the case of biochar^1^, potentially critical pollutants include heavy metals and organic pollutants such as PCBs, dioxins, or polycyclic aromatic hydrocarbons (PAHs), which can be introduced from the raw material or are generated due to the process parameters and carbonization methods [207]. According to literature [208], organic pollutants, such as pharmaceutical residues, hexachlorobenzene, or PCBs, are significantly reduced by pyrolysis. Dioxins commonly develop in combustion processes in the presence of organic C and Cl [209]. These undesirable compounds are formed at a temperature of ≥300 °C and are destroyed at ≥900 °C [ibid.]. The level of dioxins increases when higher amounts of Cl are contained, e.g., as NaCl or polyvinylchloride [210, 211]. Dioxins are often produced during waste or sewage sludge incineration, which often contain high amounts of Cl. Therefore, no or only minor dioxin contamination is to be assumed in the production of biochar from materials with a comparatively low Cl content [211] and because pyrolysis itself is not a combustion process, since there is no available oxygen. For materials with a comparatively high Cl content, it is still under research if Cl ions are volatilized in the pyrolytic process or not. The fact that Cl is enriched in fecal biochar indicates that Cl ions largely stay in the biochar [31]. Although heavy metals can accumulate during carbonization, their amounts depend strongly on the source material [209], which therefore requires low heavy metal concentrations. PAHs, which are also contained in coal and crude oil products, tobacco smoke, exhaust fume deposits, and smoked fish or grilled meat, have been shown to be carcinogenic and particularly persistent in the environment, making them a serious environmental pollutant [207, 208]. PAHs can occur in biochar due to their formation during carbonizing pyrolysis, although they are mainly found in pyrolysis gas [207]. PAH formation during pyrolysis depends highly on the process conditions, including temperature, retention times, or composition and volume of synthesis gas. At high temperatures, a large part of the plant-based feedstock is converted to gas, increasing the probability of PAH formation [207, 212]. At low temperatures, the gases condense, causing PAHs to be deposited on the coal [207, 211]. The exact conditions for PAH formation during biochar production are not yet fully understood [207, 211]. The results from our literature research suggest that a process temperature of 600–900 °C will keep the formation of PAHs at a minimum, and additional gases produced during solid pyrolysis should be burned, preventing gas and PAHs from condensing and settling on the coal [207]. Furthermore, during the cooling process after pyrolysis, the heat should be extinguished in such a way that the hot water vapor produced passes through the solid C to “clean” it (similar to the production of clean activated C). It is essential to ensure that “clean” technologies are used for carbonization and that pyrolysis occurs under controlled conditions. Modern carbonization processes can maintain very low PAH concentrations, e.g., medical and feed purposes, with no eco-toxicological potential [213]. It can therefore be assumed that it is possible to produce biochar for use as soil amendment that complies with the legal regulations regarding the pollution levels in fertilizers or sewage sludge [214]. The European Biochar Certificate (EBC) is used to guarantee and control that biochar has a sufficiently low pollutant content, to provide transparent and traceable proof of quality and to ensure sustainable production [215]. Many biochars comply with the limits for heavy metals, dioxins, or PAHs according to the EBC and DüMV [211, 216]. It was further shown that also biochar produced in modern micro-carbonization units, often used for cooking, meets the EBC criteria [217].

The operation of DTs also includes cleaning, for which detergents, odor-neutralizing substances, and fragrances are used. These may contain surfactants, phosphates, phosphonates, enzymes, optical brighteners, and silicones [218]. Particularly when collecting urine, e.g., in waterless urinals, the contamination of urine with these detergents and fragrances cannot be avoided and must be considered when assessing the risk of using urine-based fertilizers. Many cleaning and hygiene products contain quaternary ammonium compounds (QACs), which are antimicrobial chemicals used in households, industry, and livestock farming [219]. QACs are released into the environment via wastewater, sewage sludge, and manure and are associated with the spread of antibiotic-resistant bacteria [ibid.]. Fragrances, which can have an allergenic effect [220], must be listed as ingredients in detergents if threshold values are exceeded. Other adverse effects include carcinogenicity (e.g., estragole and methyleugenol) and genotoxicity (e.g., musk ketone) [221]. If fragrances enter the aquatic environment, they can cause an *anthropogenic info-chemical effect*, influencing chemical communication and therefore also the behavior and interactions of organisms in the ecosystem [ibid.]. However, little is known about the exact impact to date, making it difficult to assess the associated risks of this info-chemical effect of fragrances. Studies on the degradation of various fragrance compounds in sewage treatment plants have shown that they are completely eliminated [222]; other studies detected synthetic fragrances in European waters [223, 224]. Musk compounds on the other hand are persistent and can accumulate in organisms [225–227].

As the composition of the compost heap for composting DT contents usually consists of 1/3 feces and 2/3 additives (by volume), the quality of the additives alone is of high relevance. Of those materials included during the composting of solids from DTs, green roadside waste and biochar in particular pose a potential harmful risk associated with pollutants. The risks associated with biochar have been discussed above. Care must be taken in composting to ensure that only low-impurity biochar is used. In the case of roadside greenery, an increased concentration of heavy metals must be assumed, and are affected by their distance from the road and the volume of traffic [228]. The heavy metal contents that can be found in plants (particularly herbaceous and woody plants) depend primarily on the concentrations in the soil (geogenic loads), the soil properties, and the maintenance measures and their frequency. Pollutants can also reach the vegetation through wet or dry deposition and stick to bark or leaf mass. However, long-term testing of roadside greenery indicates that the BioAbfV impurity limits have rarely been exceeded, and that agricultural use of green roadside waste is therefore generally permissible [ibid.]. In the case of greenery alongside railways, an increased risk of pollutants must be taken into account. In addition to the basic geogenic loads of the soil or railway track ballast, heavy metals may be deposited on the railside green from the abrasion of rails and wheels, or be stored in the biomass during plant growth. It is also possible that impurities are resuspended and deposited on plants when polluted goods are being transported [ibid.].

Finally, additives may also be used in the processing of human urine into recycling fertilizers. Struvite, for example, is a crystalline compound produced by the dosage of magnesium to stored urine. By adding Mg salts (MgCl_2_, MgSO_4_, or MgO), Mg_2_^+^ reacts with PO_4_^3−^ and NH_4_^+^ to form struvite (i.e., MgNH_4_PO_4_·6H_2_O). The struvite crystals are then recovered by sedimentation or filtration [229]. As an alternative to pure Mg salts, wood ash can also be used as a Mg source [230]. While using wood ash can reduce the ecological footprint of the precipitant, heavy metals contained in wood ash might be integrated in the struvite product [ibid.]. For the abiotic stabilization of urea in urine, pH regulators are used, such as calcium hydroxide increases the pH to values around 12.5, at which the hydrolysis of urea to NH_4_^+^ by the enzyme urease is inhibited [54]. Urea hydrolysis can also be inhibited by creating an acidic environment through adding acids (e.g., citric or sulfuric acid) [231]. Urine treatment processes can further involve intensified nitrification through pH regulation with shell limestone, which is used to provide sufficient alkalinity to convert as much NH_4_^+^ as possible into NO_3_^−^ [22]. Lacto fermentation offers a biotic form of urea stabilization by acidification. During this process, lactic acid is produced, and at the resulting low pH values urea hydrolysis is prevented [, 159]. To initiate lacto fermentation, fresh urine is mixed with sugar or molasses and lactobacillus. Within a few days, the lactobacilli convert the sugar into lactic acid, reducing the pH to values below 4 [232]. In this way, the urea in the urine can be preserved for months. Sugar and molasses are known to be environmentally harmless. To sum up, by adding any chemicals to urine, good care must be taken that no critical impurities are transferred to the final fertilizer product. Moreover, when applying recycling fertilizers produced by urea stabilization via pH regulation, attention must be paid to the plant availability of the nutrients. A dilution to mitigate the strongly alkaline or acidic effect of the fertilizer may be necessary before applying the fertilizer to the soil.

#### Horticultural Suitability of Operational Additives

As with human excreta, the content and availability of nutrients essential for plant nutrition as well as the contribution of C to humus formation are the main factors in the evaluation of the horticultural suitability of operational additives. Table 7 summarizes the nutrient concentrations in human urine and feces compared to those in food waste, sawdust, and biochar. Possible horticultural risks result mainly from pollutants and the phytosanitary risks discussed above.

**Table 7:** Nutrient contents in human urine and feces and in additives used for composting. The values are given as mean ± standard deviation determined from the data in the sources. Where source FM values were converted to DM, Gaussian error propagation was applied

Material flow	C	N	NH_4_^+^-N	P	K	Mg	Ca
	g L^−1^ FM for urine, or g kg^−1^ DM for all other materials						
Urine [4, 8, 11, 44, 58, 60, 125, 126]	7.3±0.7 (n=2)	7.1±3.5 (n=22)	0.6±0.5 (n=6)	0.8±0.6 (n=15)	1.5±0.7 (n=14)	0.06±0.05 (n=6)	0.10±0.09 (n=6)
Feces [8, 44, 126, 127]	370±110 (n=1)	28.2±8.0 (n=8)	n.d.	7.8±2.9 (n=14)	10.3±4.0 (n=15)	3.4±2.3 (n=5)	17.7±9.4 (n=7)
Food waste (generic) [63, 125, 161–164]	440±63 (n=1)	22.3±7.4 (n=12)	n.d.	3.7±1.1 (n=12)	8.7±4.8 (n=14)	1.8±0.9 (n=13)	15.7±9.0 (n=13)
Sawdust (generic) [126]	488±4 (n=26)	2.6±0.5 (n=21)	n.d.	0.44±0.09 (n=7)	n.d.	n.d.	n.d.
Biochar (generic) [126]	735±11 (n=75)	3.7±0.5 (n=69)	n.d.	2.0±1.5 (n=16)	n.d.	n.d.	n.d.

*DM* dry matter, *FM* fresh matter, *n.d.* no data

In terms of toilet-related additives, toilet paper consists mostly of cellulose, and thus contains mainly C and is low in nutrients. Sawdust and biochar can also be counted among the C-rich additives with a high C content of ~50 and 75% of the DM, respectively, in comparison to human feces and food waste, with about 35% and 45% of the DM, respectively [125, 126]. Biochar, both a toilet-related and a process-related additive, is used here as a collective term for C-rich, charred organic materials, used as soil conditioners [217, 233]. In the past, biochar was an essential element of soil fertility management and was used worldwide [234]. The most “famous” soil, characterized by a high proportion of biochar, is the exceptionally fertile *Terra Preta* soil, found in the Amazon basin [235]; similar soils exist in Oceania [234] and in West Africa [236], which raised awareness of biochar as soil amendment. Depending on the soil and climate conditions, biochar decomposes slower than other organic materials and contributes to stable C build-up in the soil [237]. This material can also improve nutrient and water availability by altering the chemical and hydraulic properties of the soil and it can counteract acidification of soils due to its alkaline nature and liming effect [237, 238]. In addition, it can positively affect soil microbial activity and root symbionts, such as mycorrhizal fungi [239–241]. Biochar alone does not provide sufficient nutrients for plant growth [233] and it is therefore necessary to mix it with other organic, nutrient-rich waste such as kitchen and harvest residues or human feces. The most promising approach to soil improvement with biochar is to use it as a compost additive [242]. It has been reported that biochar withholds NO_3_^−^ and PO_4_^3−^ and, thus, become “loaded” with nutrients [242–244]. Numerous pot and field experiments and meta-analyses have confirmed that the addition of composted biochar promotes plant growth [149, 245–255].

Regarding process-related additives, when composting solids from DTs, kitchen waste and food leftovers, green waste, straw or other harvest residues, biochar, ash, or clay are normally added. The addition of these materials supports the composting process by balancing (i) nutrient-rich and C-rich material, (ii) organic fractions that are readily degradable and those that are more stable and contribute to humification, and (iii) dry and moist materials [51, 153, 256]. This combination positively influences the decomposition process and improves the quality of the compost [185]. Materials such as kitchen waste and food leftovers further contribute nutrients and moisture; green waste and biochar increase the compost C content; clay contributes minerals, i.e., secondary nutrients and trace elements important for plant growth; and green waste, straw, and biochar improve the aeration in the composting material due to their physical structure [ibid.].

### Risks Associated with Contaminants in DTs

Contaminants in DTs include waste such as hygiene products, packaging material, and glass, as well as contents from chemical toilets. Portable chemical toilet “cassettes” are widely used in mobile homes, caravans, or allotments. It is not uncommon that portable toilet contents are disposed of in public water toilets or DTs at major events, motorway service areas, camping sites, allotment gardens etc., and so the risks of contaminants introduced with the content from chemical toilets must also be considered here. The potential risks are discussed in the following section.

#### Epidemiological Risk of Potential Contaminants

Sanitary products themselves, such as wet paper, tampons, bandages, and nappies, do not pose an epidemiological risk if manufactured properly. However, due to cross-contamination with human excrements and/or blood and their associated pathogens hygiene products must be considered a source of pathogens and treated accordingly (cf. “Epidemiological Hygiene of Human Excreta” section), if they are disposed of together with excreta in DTs. Similarly, the contents of chemical toilets contain human excrements, resulting in a risk to human health. In chemical toilets, complete disinfection or hygienization is not fully achieved by the added sanitary fluids [194]. Since chemical toilets contain harmful substances (see following section), their content must *not* be processed together with the contents of DTs.

#### Pollution Characteristics of Contaminants

Contents from chemical toilets are a significant source of harmful substances. So-called sanitary liquids are added to their collection containers in order to (i) inhibit biological putrefaction processes, as well as gas and odor formation; (ii) mask the feces with perfume and color; and (iii) promote the decomposition of the feces and toilet paper [194]. The majority of sanitary fluids contain biocidal ingredients, which “are intended to destroy, deter, render harmless by chemical or biological means harmful organisms such as insects, microorganisms and rodents’ [195]. These ingredients inhibit or kill anaerobic bacteria in the feces tank, which would otherwise trigger the formation of hydrogen sulfide, mercaptans, or NH_3_ [194]. Formaldehyde, paraformaldehyde, glutaraldehyde, glyoxal, or QACs (see “Pollution Characteristics of Operational Additives” section) are used as biocidal agents. Alcohols (particularly n-propanol) may be added to these biocides to increase their effectiveness. The biocidal effect of the sanitary liquids from chemical toilets can reduce or even completely stop the biological purification stage in sewage treatment plants, if present in excessive amounts [194]. In addition to dyes and fragrances for masking the odor and appearance of feces, surfactants are also used to reduce surface tension or as emulsifiers [194]. The substances mentioned are associated with the risks described above for pollutants in detergents (“Pollution Characteristics of Operational Additives” section). Many of the substances mentioned also have a strong irritant effect on the human respiratory tract, and skin contact can cause burns and allergies. Furthermore, it is suspected that the volatile formaldehyde is carcinogenic [196]. Alternatives to the chemical sanitary liquids include sanitary additives based on enzymes that cause an aerobic, and thus largely odorless, decomposition of feces, and therefore prevent anaerobic decay processes. These act relatively slowly and require additional oxygen supply through aeration or chemical oxidants [194]. Although these enzyme-based sanitary additives pose a significantly lower risk to humans and the environment, they remain less common than biocide-based chemical sanitary liquids. To prevent the contamination of DTs with contents from chemical toilets, information for users, conspicuous labeling on DTs, and alternative disposal options for chemical toilet contents are necessary, along with a campaign recommending or prescribing the use of biodegradable products. Clear operating instructions and alternative disposal options must be installed to ensure that as few contaminants as possible reach the collection containers of DTs.

#### Horticultural Suitability of Contaminants

Hygiene products, but also packaging waste, often end up in the DTs due to misuse. These materials are largely non-biodegradable, but most could be removed by screening the raw materials before composting and additional screening and sieving of the finished compost [197]. Glass can also end up in the solid waste collection containers of DTs due to incorrect disposal. This glass, which is already shattered when it enters the toilet or in the course of the recycling process, poses a risk of injury during the horticultural use of recycled substrates. In order to avoid injuries to harvesters or loss of quality of the harvested material, only composts that have undergone fine sieving (0–10 mm) and are largely free of extraneous materials should be used in vegetable growing [198]. For this reason, proper education and operating instructions for users of DTs are vital to avoid the inclusion of incorrectly disposed materials and to ensure the production of high-quality recycling fertilizers. Due to the high levels of substances harmful to the environment (see previous section), contents from chemical toilets are not suitable for use in horticulture and must not be mixed with DT contents.

## Conclusions

Here we summarize the results of our qualitative risk analysis for the four risk categories (i) epidemiological hygiene, (ii) phytohygiene, (iii) low pollution of the environment, and (iv) horticultural suitability.

(I) Regarding the potential to cause infections, feces pose the most serious risk to human health, as it contributes the highest load of microorganisms including human pathogens. Urine is the least critical, but due to cross-contamination with feces, also source-separated urine has to be treated in order to inactivate pathogens. The risk associated with blood and vomit can be considered to be low, because the mass-related contribution to DT contents is small. However, both excreta can contain considerable amounts of pathogens and good care must be taken when handling the raw material. An effective inactivation of pathogens must be an integral part of any treatment process to reduce the risk of transmitting pathogens from human excreta including cross-contaminated toilet paper. There are various established hygienization processes for organic waste or wastewater that can also be used in the treatment of DT contents. Moreover, the most critical transmission pathway of pathogens is most probably the contact with excreta during collection and handling. It is therefore of utmost importance that established guidelines for excreta and wastewater handling are followed. With respect to human health, the risk of chemical substances must also be considered. The most critical substances are pharmaceutical residues. These substances can be degraded in the environment, but those remaining in water, soil, or fertilizer can be absorbed by plants. The likelihood of pharmaceutical uptake by plants varies according to the compound, environmental conditions, and plant species. Due to the very low levels found in crops to date, the health risk for humans is considered low; but a complete risk assessment is not yet possible, due to the low availability of relevant data.
(II) Phytohygiene is primarily relevant for recycling fertilizers, in which plant additives are included during production. The most important such fertilizer is compost. The treatment process must ensure the elimination of phytopathogens, weed seeds, and plant parts able to sprout, which are hazards to the crops being fertilized. This elimination must be verified, using existing phytohygiene regulations (e.g., the German BioAbfV) as a guide. Heat treatment is a suitable process to ensure phytohygiene in large-scale composting facilities. In the case of small-scale private composting, sanitization conditions are not easily achievable, but the associated impact is small. Nevertheless, for private composting, it is recommended that suspected phytopathogens, weed seeds, and plant parts able to sprout are disposed of with the general waste.
(III) Pharmaceuticals are the most critical substance group when it comes to pollution of the environment with impurities in human excreta–based fertilizers. However, the exact potential environmental risk posed by pharmaceuticals is still unclear. As a precautionary measure, fertilizers produced from human excreta should be analyzed for pharmaceuticals. Suggestions for indicator substances can be taken from existing guidelines for wastewater analysis. A targeted elimination of pharmaceuticals from separately collected urine, e.g., by means of activated carbon filtration, is technically possible. On the other hand, the risk of environmentally harmful heavy metal and microplastic pollution is low, due to the very low concentrations in excreta and the avoided mixed collection with different wastewater streams. Moreover, source-separation of material flows can help to reduce the exposure of the aquatic environment to pharmaceuticals, as they are not diluted with flushing water before treatment, reducing the quantitative mass/volume flow to be treated and allowing for a clearer localization of the substances to be eliminated and an appropriate targeted treatment of contaminated materials. Furthermore, common toilet- or recovery process-related additives are potential sources of pollutants, such as heavy metals or PAHs. Toilet paper produced using the latest technology poses a low risk. Toilet paper printed with heavy metal ink or mixed with perfume and disinfectants is not recommended. Wet wipes should not be disposed of in the toilet, because they are not biologically degradable. A further harmful risk arises from non-biodegradable contaminants that enter DTs through user misconduct. Wrongly disposed tampons, sanitary towels, nappies, and packaging waste can usually be removed by screening. Wrongly disposed glass poses a risk of injury during the horticultural use of recycling fertilizers and must be completely removed by fine sieving. Contents from chemical toilets can lead to pollution with formaldehyde, benzyl-C12-16-alkyldimethyl compounds, and chlorides and should definitely be kept separate from DTs. To avoid contamination with extraneous materials and contaminants at the source, we strongly recommend (a) waste bins for wet wipes and hygiene products installed inside DTs with impeded access for the user and (b) extra collection points for contents from chemical toilets with clear and conspicuous operating instructions. Possibly, adequate supervision and control are necessary.
(IV) Finally, human urine or feces are well suited nutrient-rich raw materials for fertilizer production and their positive fertilizing effect is well established. Urine-based recycling fertilizers usually have a balanced N to K ratio and a high nutrient availability. N in particular is readily available resulting in a good short-term fertilizing effect. On the other hand, feces-based fertilizers are rather suitable as P-fertilizers contain more Ca and Mg than urine, and are appropriate for humus regeneration. Feces-based compost are thus more suitable as long-term fertilizers releasing nutrients for following cropping seasons. Operational additives such as toilet paper, sawdust, or biochar do not contribute many nutrients, but they are significant sources of C, and thus play a role in humus build-up. Potential risks when using urine-based recycling fertilizers are the high salt content and NH_3_ volatilization. Increased salinity can have negative effects on soil and plants, and so it is recommended to check the soil’s salt content regularly. However, the negative effects can be offset in different ways depending on the cultivation system, such as soil-based systems or hydroponics. NH_3_ volatilization is possible, when urine is not treated before application. Biological acidification with the nitrification process is a resource-efficient process to lower the pH value and thereby strongly reducing or even eliminating the risk of NH_3_ volatilization or NH_4_^+^ toxicity. As with any fertilizer, determining the correct application rate according to plant requirements is important, to avoid nutrient losses and related environmental pollution.
  In conclusion, the benefits of using DT contents to produce fertilizers for use in horticulture are unquestionable. Certain risks to humans, soil, plants, and the environment in general exist to varying extents among the substance groups contained in DTs. However, these risks can be mitigated through awareness and education and appropriate technologies for fertilizer production and application.

## Appendix

### Indicator substances for monitoring environmentally relevant pharmaceutical residues

According to Bergmann et al. [82], the following groups of active pharmaceutical ingredients are particularly noteworthy with regard to possible negative environmental effects:

- Antibiotics
- Psychotropic drugs
- Cytostatic
- Drugs with hormonal effects (sex hormones, cytostatic hormones)

Environmentally relevant pharmaceuticals include in particular drugs, of which the consumption has risen significantly in recent years and new drugs were introduced to the market. According to Schulte-Oehlmann et al. [257], the following active ingredients should receive special attention in this respect:

- analgesics: flupirtine, ibuprofen, metamizole, naproxen;
- antibiotics: sulbactam, cefuroxime-axetil, piperacillin, ceftriaxone, levofloxacin, clindamycin, ciprofloxacin;
- antiepileptic drugs: levetiracetam, quetiapine, oxcarbazepine;
- beta-blockers: bisoprolol, metoprolol;
- lipid reducers: simvastatin;
- X-ray contrast agents: iobitridol, iomeprole, iohexol, iodixanol.

However, environmentally relevant drugs also include drugs that are used in small quantities (i.e., annual sales quantities in the kg range) but contain highly effective active pharmaceutical ingredients. According to Schulte-Oehlmann et al. [257], these include:

- antineoplastic and immunomodulating substances (e.g., hydroxycarbamide, capecitabine, celecoxib),
- urogenital system and sex hormones (e.g., lutropin alfa, medroxyprogesterone, deanol, denogest, norethisterone), and
- anti-infectives for systemic administration (e.g., amoxicillin, penicillin V, piperacillin, sulfamethoxazole, clindamycin, ciprofloxacin, cefuroxine, axetil, sulbactam, cefuroxime, ampicillin, acyclovir, cefaclor, clarithromycin, metronidazole, ceftriaxone, tazobactam, and cefazolin)

Table 8 gives an overview of some environmentally relevant active substances in human medicinal products [198].

**Table 8:** Active pharmaceutical substances from drugs for human use found in surface waters in Brandenburg [257]. The environmental relevance of the substances was determined according to the PEC:PNEC concept of Hanisch et al. [114]

Drug class	Active substance(s)
Antibiotics	Ciprofloxacin, Clarithromycin
Analgesics/Antiphlogistic	Indomethacin, Naproxen, Propyphenazone
Anesthetic	Lidocaine, Prilocaine
Antidiabetic	Metformin
Antiepileptic drugs	Carbamazepine, Primidone
Calcium antagonist	Verapamil
Lipid reducer	Bezafibrate
Lipid lowering metabolites	Clofibric acid, Fenofibric acid
X-ray contrast medium	Amidotrizoat, Iodixanol
Spasmolytics/Vasodilators	Naftidrofurylhydrogenoxalate, Pentaerythrityletranitrate
Sex hormones and analogues	Ethynylestradiol, Levonorgestrel, Medroxylprogesteronacetat, Norethisterone
Cytostatics	Cyclophosphamid, Ifosfamid, Fluorouracil

In a prioritization concept by Konradi et al. [110], four human drug substances were identified as indicator substances for sewage sludge on the basis of their occurrence and environmental behavior, namely:

1. ciprofloxacin,
2. carbamazepine,
3. clarithromycin, and
4. ethinylestradiol.

In a data collection for P-recyclates from sewage sludge by the German Environment Agency [109], the list of indicator substances to be analyzed in a drug screening (cf. [110]) was supplemented by further substances and includes the substances shown in Table 9.

**Table 9:** Pharmaceutical substances as indicator substances in sewage sludge analyses [109]

Drug class	Substance
Fluoroquinolone antibiotic	Ciprofloxacin, Levofloxacin
Macrolide antibiotic	Clarithromycin
Psychopharmaceutic	Carbamazepine
Hormone	17-α-Ethinylestradiol
Analgetic	Diclofenac
Β-Lactam-antibiotic	Cefuroxim
Sulfonamide antibiotic	Sulfamethoxazole
Hormone	17-β-Estradiol
Beta blocker	Metroprolol
Lipid reducer	Bezafibrate

A comparable assessment concept for organic trace substances from municipal wastewater was also commissioned by the Swiss Federal Office for the Environment (FOEN) [258]. It is interesting to note that the Swiss Water Protection Act (Art. 61a) requires the elimination of organic trace substances in wastewater treatment plants as early as 2016. Indicator substances are used to test the cleaning effect in wastewater treatment plants (814.201.231: DETEC Ordinance on the testing of the cleaning effect of measures for the elimination of organic trace substances in wastewater treatment plants). The substances listed in the Ordinance, Article 2, are divided into two categories:

Category 1 = substances that can be eliminated *very easily*:

- Amisulpride
- Carbamazepine
- Citalopram
- Clarithromycin
- Diclofenac
- Hydrochlorothiazide
- Metoprolol
- Venlafaxine
  Category 2 = substances that can be *easily* eliminated:
- Benzotriazole
- Candesartan
- Irbesartan
- 4-Methylbenzotriazole and 5-methylbenzotriazole as a mixture.

At least six substances must be examined for the calculation of the cleaning effect. “The ratio of substances in category 1 to substances in category 2 must be two to one.” (VO 814.201.231 Art. 3). Some of the indicator substances used in Switzerland were also used in the UBA data collection (Table 4): carbamazepine, diclofenac, and metoprolol [109].

### The German Federal Environment Agency’s database on pharmaceuticals in the environment

The following search criteria were applied to the data on sewage sludge, soil, and liquid manure from the UBA database [105] contained in Table 4:

- Sampling Country: *EU countries*, *including Switzerland and Norway*
- Credibility of the data, or the quality of the data (“Literature Credibility”): *good*
- Detection of a substance: *only positive results*
- Sample matrix (“matrix”):
- Sewage sludge (all *WWTP sludge* fractions and *sewage sludge*, no wastewater from sewage treatment plants) and resulting database entries n=1 117
- Liquid manure (*manure liquid*) n=122
- Solid manure (*manure dung*) was not shown in Table 4 for simplification, as it was largely reflected in the values of liquid manure, n=50
- Soil, n=330
  - To simplify matters, maximum contents of the standardized values of the measured environmental concentration (MEC) were used.
  - The individual values taken from the UBA database have different units (see table below). For comparability with Bergmann et al. [82], the values in Table 4 were given in μg kg^−1^ FM or DM, or μg L^−1^.
  - Table 4 contains only a selection of data based on indicator substances and the largest possible intersection of data in the different matrices in direct comparison.

The individual values taken from the UBA database have different units: μg L^−1^, μg kg^−1^ of DM, μg kg^−1^ of FM or μg kg^−1^ without specification of DM or FM, see the following summary in Table 10.

**Table 10:** Number of data entries (n) derived from the database [105] per respective unit in three different matrices

Matrix	Total (n)	μg L^−1^	μg kg^−1^ DM	μg kg^−1^ FM	μg kg^−1^ only
Sewage sludge	1 127	56	19	336	716
Slurry (liquid manure)	122	37	6	–	79
Soil	330	4	26	3	297

## Footnotes

1 Here, “biochar” refers to coal produced by pyrolysis of plant material, including wood, green cuttings, and crop residues; the use of coal produced by hydro-thermal carbonization (“hydrochar”) is not covered herein.

## Abbreviations

AOX: Adsorbable organic halogens
BGK: Federal Compost Quality Association (*Bundesgütegemeinschaft Kompost)*
BioAbfV: German Biowaste Ordinance (*Bioabfallverordnung*)
BPA: Bisphenol A
CE: Circular economy
DIN: German Institute for Standardisation (*Deutsches Institut für Normung*)
DIN SPEC: DIN product specification
DM: Dry matter
DT: Dry toilet
DWA: German Association for Water Management, Sewage and Waste

## (*Deutsche Vereinigung für Wasserwirtschaft, Abwasser und Abfall*,

DüMV: German Fertilizer Ordinance (*Düngemittelverordnung*)
DüV: German Fertilization Ordinance (*Düngeverordnung)*
EHEC: Enterohaemorrhagic *Escherichia Coli*
FM: Fresh matter
GHG: Greenhouse gas
HIV: Human immunodeficiency virus
MAP: Magnesium ammonium phosphate
PAH: Polycyclic aromatic hydrocarbons
PCB: Polychlorinated biphenyls
PEC: Predicted environmental concentrations
PNEC: Predicted no effect concentrations in soils
QAC: Quaternary ammonium compounds
RQ: Risk quotient
UBA: German Federal Environment Agency (*Umweltbundesamt)*
WHO: World health organization

## Chemical elements and molecules

Ca: Calcium
Cd: Cadmium
CH_4_: Methane
CH_4_N_2_O: Urea
Cl: Chloride
Cr: Chromium
Cu: Copper
K: Potassium
Mg: Magnesium
N: Nitrogen
N_min_: Mineral N
N_org_: Organically bound N
NH_3_: Ammonia
NH_4_^+^: Ammonium
NO_3_^-^: Nitrate
N_2_O: Nitrous oxide
NaCl: Sodium chloride
Ni: Nickel
P: Phosphorus
PO_4_^3-^: Phosphate
Zn: Zinc
